# Evaluating the Contribution of *Nocardia* spp. and *Mycobacterium tuberculosis* to Pulmonary Infections among HIV and Non-HIV Patients at the Komfo Anokye Teaching Hospital, Ghana

**DOI:** 10.1155/2018/2910198

**Published:** 2018-11-19

**Authors:** Samuel Asamoah Sakyi, Kwabena Owusu Danquah, Richard Dadzie Ephraim, Anthony Enimil, Venus Frimpong, Linda Ahenkorah Fondjo, Esther Love Darkoh

**Affiliations:** ^1^Department of Molecular Medicine, School of Medical Sciences, Kwame Nkrumah University of Science and Technology (KNUST), Kumasi, Ghana; ^2^Department of Medical Laboratory Technology, Faculty of Allied Health Sciences, Kwame Nkrumah University of Science and Technology (KNUST), Kumasi, Ghana; ^3^Department of Medical Laboratory Technology, School of Allied Health Sciences, University of Cape Coast (UCC), Cape Coast, Ghana; ^4^Department of Child Health, School of Medical Sciences, Kwame Nkrumah University of Science and Technology (KNUST), Kumasi, Ghana; ^5^Department of Theoretical and Applied Biology, College of Science, Kwame Nkrumah University of Science and Technology (KNUST), Kumasi, Ghana

## Abstract

Tuberculosis (TB) is a major cause of human mortality particularly in association with the human immunodeficiency virus (HIV). *Nocardia* spp. has emerged as an opportunistic infection especially in HIV patients. The high prevalence of TB and HIV coupled with the lack of a definitive laboratory diagnosis for *Nocardia* spp. could lead to misdiagnosed pulmonary TB. This study determined the prevalence of pulmonary infections due to *Nocardia* spp. and *Mycobacterium tuberculosis* in sputum of HIV and non-HIV patients with suspected pulmonary tuberculosis at KATH. A total of sixty sputum samples were obtained from HIV and non-HIV patients with suspected pulmonary tuberculosis. Samples were examined by fluorescence based Ziehl–Neelsen staining, culture, and PCR methods. The prevalence of *Nocardia* spp. and *Mycobacterium tuberculosis* was 18.3% and 20%, respectively, with the latter having the highest rate among patients aged 21–40 years (*P*=0.075). The prevalence of *Nocardia* spp. among HIV patients was 90.9% whilst 16.7% of the patients had HIV/*Nocardia* spp. coinfection. Detection of *Mycobacterium tuberculosis* by fluorescence-based Ziehl–Neelsen staining, culture, and PCR yielded 9 (15%), 11 (18.3%), and 12 (20%), respectively. There is a high prevalence of nocardiosis especially in HIV patients. PCR is a better diagnostic method that detects both *Nocardia* spp. and *Mycobacterium tuberculosis* and should be incorporated into routine diagnosis for pulmonary infections.

## 1. Introduction

Tuberculosis remains a major cause of human mortality and morbidity, threatening the lives of one-third of the world's popu [1]. It is caused by *Mycobacterium tuberculosis* complex such as *Mycobacterium bovis*, *M. africanum*, *M. bovis bacillus* Calmette–Guerin, *and M. tuberculosis* [2, 3]. The latter, however, is responsible for most tuberculosis (TB) infections in humans [4]. An estimated 7–9 million cases of TB are reported annually resulting in 1.5–2 million deaths worldwide [5]. According to the WHO [6], TB is endemic in sub-Saharan Africa with about 1.5 million cases reported annually. This high prevalence of TB in developing countries is due to poverty, overcrowding, and HIV infection. In Ghana, the disease plaques all segments of the society and over 46,000 new cases of TB are reported annually [6]. A study conducted in Ghana on the burden of HIV on TB patients showed that 1633 (92.2%) tested positive to HIV from 1772 TB patients [7].

*Nocardia asteroides* is the most common causative agent of pulmonary nocardiosis, accounting for 85% of all cases [8]. It is contracted through inhalation into the respiratory tract. The pulmonary event in humans may either be self-limiting, transient, or subclinical, or it may progress to an acute, subacute, or chronic process mimicking tuberculous (TB) or mycotic infection or a malignancy. The infection is more common in immunocompromised hosts [9–11] especially those with impaired cell-mediated immunity such as patients with HIV infection, those on long-term corticosteroid exposure, malignancy, chronic alcoholism, and diabetes mellitus, and patients with a history of solid organ transplantation [8, 12–17].

The laboratory diagnosis and clinical manifestation of TB and nocardiosis are similar and requires accurate laboratory test in order to distinguish one from the other. The lack of a definitive laboratory diagnosis of nocardiosis has often led to misdiagnosis and hence mistreatment [18]. Additionally, cases of pulmonary nocardiosis in HIV-infected persons have also been diagnosed as pulmonary TB [19]. Moreover, little is known about the coinfection of pulmonary nocardiosis and tuberculosis in HIV and non-HIV patients in Ghana. Accurate identification of *Nocardia* species is burdened by its characteristic branching on Gram staining, which makes it seem acid fast and mimics *Mycobacterium tuberculosis*. Besides, conventional methods involve long-term culture, the identification of growth characteristics of colonies, assessment of microscopic morphology of colonies, and biochemical and susceptibility testing. This method therefore carries a high risk of contamination from other bacteria and fungi from the environment.

In as much as nocardiosis resembling tuberculosis, first-line antituberculous drugs are not efficient for its treatment. Moreover, optimal therapeutic strategies depend on rapid and accurate identification of *Nocardia* spp. As such, the use of molecular methods for identification, such as PCR with high sensitivity and specificity, is necessary to improve the accuracy of diagnosis of nocardiosis. Therefore, the suggested approach to overcome this problem is molecular techniques, since they are more specific, sensitive, and rapid as compared to conventional diagnostic methods. Consequently, unraveling the prevalence of pulmonary infections due to *Nocardia* species and *Mycobacterium tuberculosis* is important in mitigating misdiagnosis of pulmonary infections especially among HIV/AIDS patients and ultimately ensure accurate diagnosis and treatment.

## 2. Materials and Methods

### 2.1. Study Design

This hospital-based cross-sectional study was conducted at the Komfo Anokye Teaching Hospital (KATH) in Kumasi from March to July, 2017.

### 2.2. Study Area

The study was conducted at KATH which is located in the Kumasi metropolis. The metropolis is one of the 27 districts in Ashanti region, with a population of about 1,730,249. The hospital is one of the largest health facilities serving both as the first consultation point and as a referral centre for the Northern and middle belt of Ghana.

### 2.3. Ethical Considerations

Ethical approval was sought from the Committee on Human Research, Publication and Ethics (CHRPE) of the School of Medical Sciences, Kwame Nkrumah University of Science and Technology (CHRPE/AP/564/17). All participants gave their written informed consent after the aim and objectives of the study had been explained to them.

### 2.4. Inclusion and Exclusion Criteria

HIV and non-HIV patients suspected with pulmonary tuberculosis with laboratory request by clinicians to undergo pulmonary tuberculosis investigations were recruited as subjects. However, HIV and non-HIV patients without any clinical suspicion of pulmonary infection were excluded. A total of 60 patients aged between 4 and 80 years were recruited into the study.

### 2.5. Sputum Collection and Questionnaire Administration

Structured questionnaires were administered to all study participants to collect demographic information and medical history and HIV infection status as well as information on the use of steroids and previous transplantation history. Two milliliter of early morning and on-spot sputum specimens were collected from each participant with suspected pulmonary tuberculosis. The participant was requested to cough so that expectoration comes from as deep down the chest as possible and spat into a sterile, wide mouth, and leak-proof specimen containers.

### 2.6. Sputum Decontamination and Processing

The obtained sixty sputum samples were decontaminated immediately according to standardized routine diagnosis procedures by the NaOH/N-acetyl-L-cysteine (NALC) method. The concentrated specimen was then used for culture, smear preparation, and PCR assays for *Mycobacterium tuberculosis* and *Norcadia* spp. [20]. For the culture, 250 *µ*l of each of the concentrated sputum was inoculated onto Löwenstein–Jensen media and incubated at 37°C. *Nocardia* spp. are acid fast, can withstand the decontamination with NaOH as well as NALC method, and thereby can grow on Löwenstein–Jensen media. Colonies that were suspicious were examined using acid-fast and Gram-staining method while cultures that did not show any growth after some weeks were reported as negative.

Prior to microscopic examination, a drop of the sediment of the sputum sample was spread on each clean microscopic slide using a Pasteur pipette. Slides were allowed to dry, fixed by heat, and stained using Ziehl–Neelsen acid-fast staining.

### 2.7. DNA Extraction and PCR Assay

After decontamination, the genomic DNA was extracted using GenoLyse Kit (VER 1.0, Hain Lifesciences, Germany) following the manufacturer's instructions. Primers for *Norcadia asteroides* ATCC 19247 and *Mycobacterium tuberculosis* H37RV were synthesized and purchased from inqaba biotec, South Africa. The isolated DNA was amplified using specific pairs of primers IS1 (5′CTCGTCCAGCGCCGCTTCGG3′) and IS2 (5′CCTGCGAGCGTAGGCGGTGG3′) for *Mycobacterium tuberculosis* complex. The PCR protocols were optimized to 30 cycles consisting of 5 minutes at 96°C for initial heat activation, 1 minute at 95°C for denaturation, 1 minute at 65°C for annealing, and 1 minute at 72°C for extension, whereas the final extension was done for 1 minute at 72°C.

Primers NG1 (5′CTCGTCCAGCGCCGCTTCGG3′) and NG2 (5′CCTGCGAGCGTAGGCGGTGG3′) were used to amplify a *Nocardia* genus-specific 590 bp fragment of 16S rRNA. The PCR protocols were optimized to 30 cycles consisting of 10 minutes at 94°C for initial denaturation, 1 minute at 94°C for denaturation, 20 seconds at 55°C for annealing, and 1 minute at 72°C for extension. The final extension was done for 10 minutes at 72°C.

**Figure 1:**
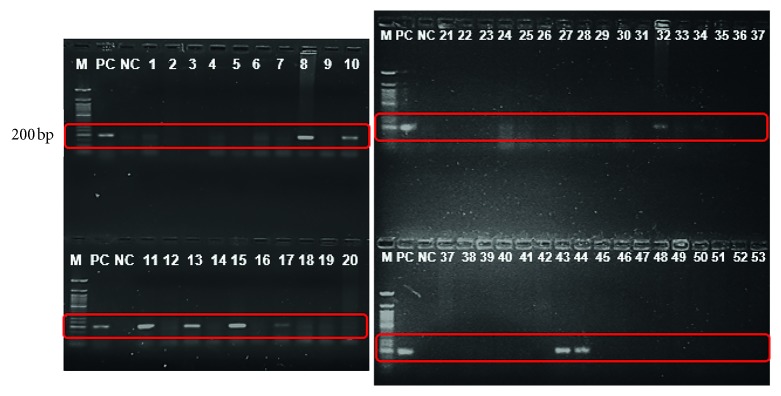
Amplification of TB with IS6110 primers. M: molecular weight marker, PC: positive control, NC: negative control, bp: base pairs (expected base pairs of *Mycobacterium tuberculosis,* 200 bp).

**Figure 2:**
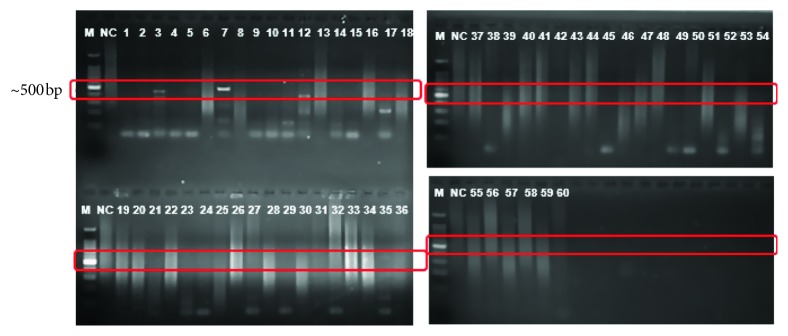
Amplification of *Nocardia* with IS1&2 primers: M: molecular weight marker, PC: positive control, NC: negative control, bp: base pairs (expected base pairs of *Nocardia asteroides*, 500 bp).

Amplification with these primers was observed by electrophoresis on 2% (w/v) agarose gel stained with ethidium bromide as shown in Figures 1 and 2. The *Nocardia asteroides* ATCC 19247 and *Mycobacterium tuberculosis* H37RV were used as positive reference strains. Two PCRs for each sample were performed in separate tubes with two pairs of primers. One set was dedicated to the *Mycobacterium tuberculosis* complex, whereas the other set was for *Nocardia* spp. For each round of PCR, ddH_2_O was used as the negative template.

## 3. Analysis

Data analysis was carried out using the Statistical Package for the Social Sciences (SPSS, version 23). The chi-square test was used to determine the significance differences between variables, and the level of significance was set at *P* < 0.05.

## 4. Results

### 4.1. Demographic Characteristics/Prevalence of Pulmonary Infections

Of the 60 participants that were recruited into the study, 56.7% (34/60) were males whilst 43.3% (26/60) were females with ages ranging from 4–80 years, whereas the mean age of the study population was 43.9 ± 19.0 years. Additionally, 25% were HIV positive whereas 75% of the participants were HIV negative (Table 1).

Prevalence of *Nocardia* spp. and *Mycobacterium tuberculosis* was 18.3% and 20%, respectively. Though males had higher number of infections caused by *Mycobacterium tuberculosis* than females, this difference was not statistically significant (*P*=0.896). Moreover, pulmonary infection caused by *Nocardia* spp. (*P*=0.087) and *Mycobacterium tuberculosis* (*P*=0.075) between ages showed no significant difference as presented in Table 2.

**Figure 3:**
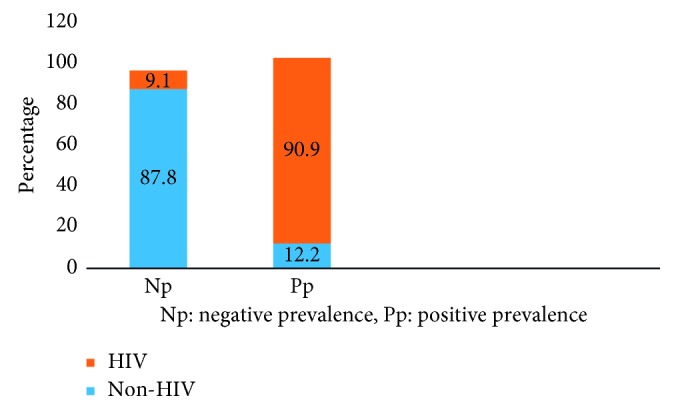
Prevalence of *Nocardia* spp. in HIV and non-HIV patients.

It was also observed from the study that the prevalence of *Nocardia* spp. was higher in the HIV patients (90.9%) as compared to the HIV-negative group (12.2%), and the difference was statistically significant (*χ*^2^=  26.181; *P*=0.000) as shown in Figure 3.

**Figure 4:**
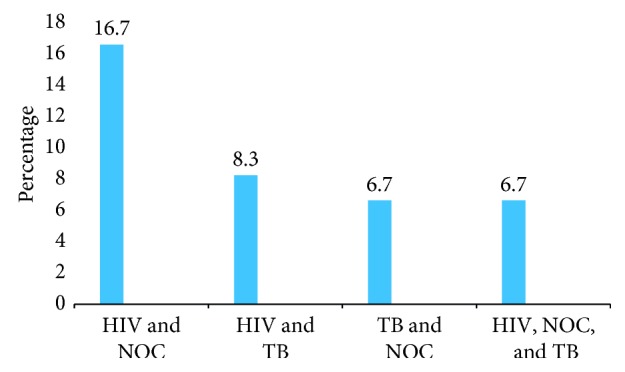
HIV, TB, and *Nocardia* spp. coinfection among study participants. NOC, *Nocardia*.

With regards to coinfection, study participants harbored two or more diseases, and of the total study sample size (60), coinfection of HIV and *Nocardia* spp. was observed in 10 (16.7%) participants while 5 (8.3%) had both HIV and tuberculosis (Figure 4).

**Figure 5:**
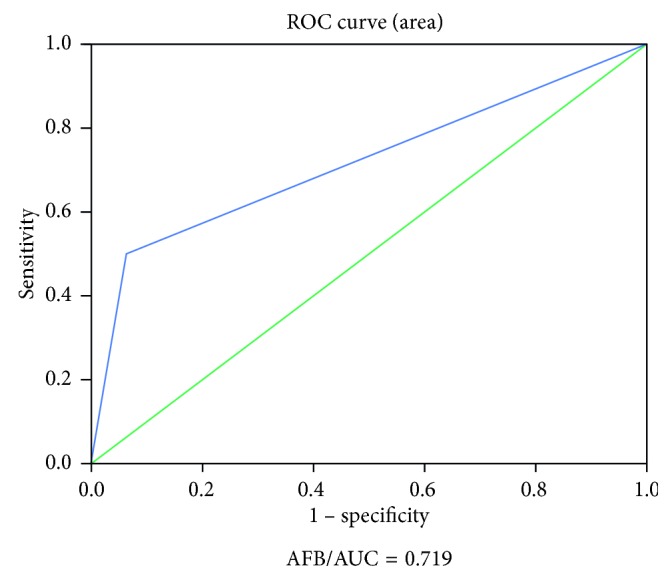
ROC of AFB in diagnosing TB.

Findings from fluorescence-based Ziehl–Neelsen examination indicated that *Mycobacterium tuberculosis* was found in 9 (15%) samples, and culture identified 11 (18.3%) positives whereas PCR analysis identified 12 (20%) positives for *Mycobacterium tuberculosis.* Moreover, based on the area under the receiver operating characteristic (ROC) curve, the use of microscopy in diagnosing TB was 71.9% (Figure 5).

## 5. Discussion

This study determined the prevalence of *Nocardia* spp. and *Mycobacterium tuberculosis* in sputum samples of patients with suspected pulmonary infections. Gender is an important factor for determining pulmonary infection according to Gupta et al. [21] and Gajbhare et al. [22]. The current study revealed that pulmonary infections were higher in males than females and were common in the age group of 21–40 years (10.6%). This affirms the findings of Codlin et al. [23] who reported higher prevalence of tuberculosis in males than in females. A related study conducted by Muniyandi et al. [24] on socioeconomic dimensions of tuberculosis control showed that 73% of the study population were males. This could be due to the fact that more males are likely to involve in activities that will predispose them to reduced immunity. Moreover, socioeconomic reasons may further subject them to poor diet and overall poor health conditions. In the developing countries, men are seen as the head of the family, and they tend to engage in various job-related activities with its associated hazards, which expose them to pulmonary infections. This study further observed that majority of the study participants that had pulmonary infections were HIV positive. This corroborates the studies by Kherad et al. [25] and Luetkemeyer [26] who also reported high coinfection in tuberculosis and HIV patients. This is due to the fact that individuals with HIV infection are immunocompromised and that predisposes them to TB infection. Contrary to the high prevalence of TB infection among HIV patients in our study, Sharma et al. [27] reported low tuberculosis infection in India. The low TB observed in this study could be due to overall low prevalence of TB in the study area.

Opportunistic microorganisms like *Nocardia* spp. can mimic pulmonary tuberculosis in HIV/AIDS and non-HIV patients and can be fatal if untreated [28]. Some investigators have previously reported incidence of pulmonary nocardiosis and tuberculosis in HIV-infected patients [16, 29]. Ekrami et al. [28] reported concomitant pulmonary nocardiosis and tuberculosis in HIV patients. It was found in their study that the coincidence of pulmonary tuberculosis and nocardiosis was 1% for their entire study population and 6.25% among HIV-infected patients. In another study, pulmonary nocardiosis in HIV-infected patients with suspected pulmonary tuberculosis was reported to be 3% by Alnaum et al. [30]. In our study, however, 90.9% of *Nocardia* spp. was from HIV patients whereas 6.7% had tuberculosis, HIV, and nocardiosis coinfection and may be attributed to suppression of cell-mediated immunity [31]. In addition, epidemiological data on the prevalence of AIDS-associated nocardiosis are scarce; nevertheless, based on initial studies from other parts of the world, nocardiosis has been regarded as an unusual complication of HIV infection. The reason for this apparently high prevalence of HIV-associated nocardiosis is unclear and probably multifactorial. Though previous studies have reported that between 57% and 68% of patients have AIDS-defining criteria at the time of diagnosis of *Nocardia* infection [12, 18]. The disease may still be underreported [32].

Diagnosis of tuberculosis using molecular techniques like the PCR has been more efficient than the conventional methods [33, 34]. In the current study, we detected 20.0% and 15% of *Mycobacterium tuberculosis* in participants with the use of polymerase chain reaction (PCR) and acid-fast bacilli (AFB) correspondingly. Nonetheless, *Nocardia* was not distinguished in sputum specimens using conventional methods in this study, but positive samples that yielded a result of 18.3% were determined using the PCR assay. The prevalence of *Nocardia* in this study was significantly lower than a prevalence of 37.5% from Dawar et al. [35] on epidemiology of nocardiosis—a six-year study from Northern India. The difference in prevalence may be due to differences in methodology, as most of the other studies used microscopy, whilst this study used polymerase chain reaction. Another reason could be due to sampling technique as this current study concentrated on patients with suspected TB whilst the other studies ranged from immunocompromised patients to patients from the general population.

This result is very significant because previous laboratory-based data had not isolated any *Nocardia* in the chest clinic of the Komfo Anokye Teaching Hospital. *Nocardia* spp. is one of the opportunistic infections in the HIV/AIDS patients just as tuberculosis is and can be fatal if untreated. Untreated pulmonary nocardiosis is very similar to tuberculosis particularly in immunocompromised patients and could be mistaken for other bacterial infections and underestimated. Also, as much as nocardiosis resembles tuberculosis, first-line antituberculous drugs are not efficient for its treatment. Moreover, optimal therapeutic strategies depend on rapid and accurate identification of *Nocardia* spp. This means that the chest clinic at the Komfo Anokye Teaching Hospital must put in measures to diagnose *Nocardia*, since it is likely that a lot more patients are being missed and runs a risk of complications due to the nontreatment.

## 6. Conclusions

The prevalence of *Mycobacterium tuberculosis* in the study population was 20% whilst *Nocardia* spp. was 18.3%. The most prevalent coinfection observed was HIV and *Nocardia* spp. with an occurrence of 16.7%. Hence, in Ghana, a better diagnostic method with high sensitivity and specificity should be employed to improve the accuracy of diagnosis of nocardiosis. Moreover, *Nocardia* diagnosis should be incorporated into routine diagnosis for pulmonary infections in patients and especially those who fail to respond to anti-TB treatment in Ghana.

## Figures and Tables

**Table 1:** Sociodemographic characteristics of study participants.

Variables	Frequency	Percentage (%)
Ages		
** **<20	7	11.7
** **21–40	18	30.0
** **41–60	25	41.7
** **≥61	10	16.7
Gender		
** **Male	34	56.7
** **Female	26	43.3
HIV status		
** **Positive	15	25.0
** **Negative	45	75.0

**Table 2:** Prevalence of *Nocardia* spp. and *Mycobacterium tuberculosis*.

Variable	Number infected	Prevalence (%)	Chi-square value (*χ*^2^)	*P* value
Infections due to *Mycobacterium tuberculosis*				
Gender				
** **Male	7	11.7	0.016	0.896
** **Female	5	8.3		
Ages				
** **≤20	1	8.3		
** **21–40	7	58.3	6.907	0.075
** **41–60	4	41.7		
** **≥61	—	—		
*Nocardia* spp.				
Gender				
** **Male	9	81.8	3.470	0.062
Female	2	18.2		
Ages				
** **≤20	—	—		
** **21–40	6	54.5	6.568	0.087
** **41–60	5	45.5		
** **≥61	—	—		

## Data Availability

The data used to support the findings of this study are included within the article.
